# Research on hybrid cloud resource scheduling optimization algorithm based on EMPA-ASA

**DOI:** 10.1371/journal.pone.0346727

**Published:** 2026-04-10

**Authors:** Zhigang Zhang, Jiaqi Gao, Rong Liu, Qibing Tao

**Affiliations:** Tianjin Chengjian University, Tianjin, China; Alma Mater Studiorum Universita di Bologna: Universita degli Studi di Bologna, ITALY

## Abstract

Hybrid–cloud scheduling must balance cost, performance, and reliability; yet existing approaches often suffer from burdensome parameter tuning, a limited set of optimized QoS indicators, and high computational overhead. To address these issues, we propose an EMPA–ASA–based hybrid–cloud resource scheduling algorithm and make three contributions: 1) we realize state-driven adaptive scheduling and resource allocation via MDP + Q-learning, updating the policy online as system conditions evolve; 2) we introduce an *M*/*M*/*c* queueing model to quantitatively encode QoS constraints, thereby improving responsiveness and load adaptivity; and 3) we fuse EMPA with Adaptive Simulated Annealing (ASA), augmented by Lévy flights to strengthen global exploration and accelerate convergence. We implement a full prototype and conduct performance evaluations. The results show that EMPA–ASA outperforms baselines across multiple QoS metrics—including end-to-end delay, response time, throughput, and packet-loss rate—and reduces total cost by approximately 48% and 70% relative to GA and PSO, respectively; its advantages in QoS and cost are especially pronounced under high-load scenarios. These findings indicate a superior cost–performance trade-off, providing an efficient and reliable solution for hybrid–cloud resource scheduling.

## Introduction

In modern IT architectures, hybrid clouds have become an effective means of optimizing resource allocation [1,2]. By leveraging the complementary strengths of private and public clouds, enterprises can flexibly place workloads to satisfy service–quality requirements while meeting performance, compliance, and cost constraints, thereby improving overall service capability. Although managing hybrid-cloud environments offers compelling advantages [3,4], achieving effective and efficient control remains challenging because of the intrinsic heterogeneity of the underlying infrastructure and the geographical dispersion of resources. Unlike homogeneous settings, a hybrid cloud spans public– and private–cloud domains with diverse virtualization stacks, storage systems, and network fabrics [5,6]. This complexity is further exacerbated by the dynamic and unpredictable latency and bandwidth of inter-cloud links. Consequently, designing a scheduling algorithm that explicitly contends with heterogeneity and complexity—so as to raise resource utilization while reducing energy consumption and operating costs—has become a central problem in hybrid-cloud computing.

Current hybrid-cloud schedulers face several key limitations. First, resource utilization is often low—especially under light loads—leading to idle capacity and waste [7]. Second, task response times remain high and jittery, which in turn inflates overall costs [8]. Third, during low-to-moderate load phases, mismatches between iteration cadence and resource provisioning frequently cause capacity waste and degrade energy efficiency [9]. To remedy these deficiencies, we propose *EMPA–ASA*. Our core innovation is to fuse Adaptive Simulated Annealing (ASA) with the Enhanced Marine Predator Algorithm (EMPA), achieving fast local convergence together with strong global exploration. The contributions of this study are threefold:

- **Proposed a dynamic congestion control and load-aware adaptive mechanism:** In our solution, we have improved a congestion control mechanism. This mechanism dynamically adjusts the scheduler based on different system loads (low, medium, and high). This ensures that the system can adapt to actual hybrid cloud operations, and improves the stability and resource utilization of the system compared to the existing system.
- **Improved the effectiveness of elastic resource allocation:** We propose a flexible resource management strategy that adapts to load variations by dynamically scaling resources. When demand decreases, resources are downscaled, and task admission is controlled. Conversely, in overload situations, the system applies backpressure to upstream tasks, redirects excess jobs to a dead-letter queue (DLQ), and prioritizes critical tasks. This adaptive approach ensures that the system can scale elastically while maintaining performance and preventing resource bottlenecks.
- **Optimized the cost-effectiveness scheduling based on the hybrid model of EMPA–ASA:** The core of our algorithm combines the Enhanced Marine Predator Algorithm (EMPA) for global exploration with Adaptive Simulated Annealing (ASA) for local refinement. This hybrid approach balances the global search for optimal solutions with the local intensification required for fast convergence. It optimizes the trade-off between multiple QoS parameters and operational costs, making it particularly effective in minimizing total system cost while ensuring that stringent QoS constraints are satisfied in hybrid-cloud environments.

The remainder of this paper is organized as follows. The Related Work section surveys research on hybrid-cloud scheduling. The Model and Formulation section details the system model and problem formulation. The Algorithm Design (EMPA–ASA) section presents the design of the proposed EMPA–ASA algorithm. The Experiment and Simulation section describes the experimental setup and discusses the results. The Conclusion section concludes the paper and outlines future research directions.

## Related work

### Resource scheduling algorithm

Current research primarily focuses on optimizing resource scheduling strategies, resource utilization, task completion time, and energy consumption in cloud platforms. Xie et al. [10] proposed an Improved Honey Badger Algorithm (IHBA), which integrates a multi-strategy local search mechanism and an optimized fitness function to enhance population diversity and global search ability, addressing the issue of traditional metaheuristic algorithms getting trapped in local optima during cloud resource scheduling. Yin et al. [11] introduced a Novel Genetic Ant Colony Optimization (NGACO) algorithm by combining an improved Genetic Algorithm (GA) and Ant Colony Optimization (ACO). This algorithm employs random initialization to enhance exploration and incorporates optimized pheromone update and penalty mechanisms to improve scheduling efficiency. Mustapha et al. [12] proposed a task scheduling algorithm based on DBSCAN clustering to optimize execution efficiency and improve service quality. Experimental results show that the method outperforms ACO and PSO in terms of execution time and overall scheduling performance. Shobeiri et al. [13] developed a hybrid scheduling algorithm, PCP-ACO, which combines the PCP heuristic for task ordering with ACO for optimal resource selection, significantly reducing workflow execution costs under cloud environments while meeting deadline constraints. Cheng et al. [14] proposed a task scheduling algorithm based on an improved A3C model, which incorporates Residual Convolutional Neural Networks (RCNN) to optimize model structure and adopts asynchronous multi-threaded training to adapt to dynamic resource changes. Experimental results show that the algorithm effectively reduces task response time and system energy consumption, while improving resource utilization and load balancing. Murad et al. [15] introduced an improved cloud job scheduling algorithm, SG-PBFS, built on a Priority-Based Fair Scheduling (PBFS) framework. By incorporating the Shortest Gap Backfilling Strategy (SG), the algorithm optimizes scheduling gaps, enhancing the performance of priority rule (PR) schedulers and increasing resource utilization. Shukla et al. [16] proposed a Differential Evolution–Grey Wolf Optimizer (DE-GWO) algorithm to improve scientific workflow scheduling efficiency in cloud–fog environments. The method accelerates the GWO convergence and improves optimization accuracy through DE, while a weighted objective function is designed to optimize makespan, cost, and energy consumption. DE introduces evolution and elimination mechanisms into GWO, and GWO maintains a good balance between global exploration and local exploitation.

Studies on resource–scheduling algorithms in [10–16] predominantly optimize policies under a *single* load condition. However, when confronted with complex and rapidly varying workload fluctuations, resource bottlenecks, or adverse network conditions, these approaches typically optimize only a limited subset of performance indicators and exhibit restricted flexibility and adaptivity in resource allocation.

To address these limitations, various multi-objective optimization algorithms have emerged recently. For instance, the enhanced multi-objective cuckoo search algorithm with migration operators [17] and the enhanced beluga whale optimization algorithm based on a ring topology structure [18] optimize multiple objectives in cloud and Internet of Things environments. The former uses the migration operator to balance exploration and exploitation in IoT task scheduling, but due to its reliance on a fixed search strategy and limited adaptability to real-time workload fluctuations, its performance may decline in dynamic hybrid cloud conditions. The latter enhances the global exploration ability through a ring topology structure, but has difficulties in real-time adaptation to variable resource availability, which is a key challenge in the hybrid cloud environment. Furthermore, the EMO-TS algorithm [19] combines deep reinforcement learning with enhanced electric fish optimization to achieve energy-efficient task scheduling in cloud data centers, significantly improving energy consumption and completion time. However, its main focus on energy optimization not fully address the joint optimization problem of multiple quality of service indicators (such as response time, jitter, and packet loss rate). Given these shortcomings, our work addresses the multi-load situation (low/medium/high) and proposes a scheduling method that can jointly optimize multiple quality of service indicators – response time, packet loss rate, end-to-end delay, and jitter – while dynamically adapting to the constantly changing conditions of the hybrid cloud environment. By combining EMPA with ASA and integrating global exploration with local reinforcement, it achieves robust real-time strategy adaptation.

### Improved simulated annealing algorithm

Liu et al. [20] proposed a cloud computing task scheduling mechanism based on the Simulated Annealing (SA) algorithm. Compared with traditional scheduling algorithms such as Genetic Algorithm (GA) and Particle Swarm Optimization (PSO), this method demonstrates significant advantages in reducing task execution delay (by 23.6%)and improving virtual machine resource utilization (by 18.9%). Moreover, the introduction of a probabilistic suboptimal solution acceptance strategy effectively avoids local optima. Mondal et al. [21] proposed a load balancing strategy for cloud computing based on SA. Compared to traditional scheduling methods such as First-Come-First-Serve (FCFS), Round Robin, and Stochastic Hill Climbing (SHC), this algorithm employs an annealing temperature-controlled probabilistic acceptance mechanism to avoid being trapped in local optima. Experiments show significant improvements in task response time and virtual resource utilization over baseline methods. Celik et al. [22] introduced a cluster-based metaheuristic task scheduling method based on SA, which reduces the execution time of developed computer programs and improves the quality of scheduling solutions. Khaledian et al. [23] proposed a hybrid Particle Swarm Optimization–Simulated Annealing (PSO-SA) algorithm for prioritizing and assigning workflow tasks in cloud-fog environments. Compared with the baseline algorithm IKH-EFT, PSO-SA improves energy efficiency and total completion time by 5% and 9%, respectively, over traditional PSO and SA algorithms. Lv et al. [24] developed a load balancing strategy based on SA (SA-LB), which utilizes an annealing temperature-controlled probabilistic solution acceptance mechanism. It exhibits notable advantages in reducing average task response time and improving virtual machine resource utilization. Kumar et al. [25] proposed a Hybrid Spider Monkey Optimization–Simulated Annealing (HSMO-SA) approach. HSMO-SA demonstrates a significant reduction in resource scheduling costs compared to standard SMO, classical SA, and PSO algorithms.

Research on improved simulated annealing (SA) for task scheduling and load balancing [20–25] has achieved notable gains; yet these methods still suffer from intricate parameter tuning, relatively slow convergence, and nontrivial computational overhead—limitations that become more pronounced in dynamic computing environments. To address these issues, we couple EMPA with ASA and tightly integrate them with the proposed QoS- and congestion-aware mechanisms, enabling policy adaptation and rapid convergence under real-time workload fluctuations. As a result, our approach reduces total cost while simultaneously improving multiple indicators, including response time, throughput, latency, and jitter.

### Improved EMPA algorithm

Gong et al. [26] proposed an Enhanced Marine Predator Algorithm to improve scheduling efficiency, aiming to minimize task completion time and enhance resource utilization. Saravanan et al. [27] introduced an Improved Wild Horse Optimizer with Lévy Flight (IWHOLF-TSC), which shows significant advantages over traditional scheduling algorithms in reducing task execution time and improving resource utilization. Bi et al. [28] proposed a computation offloading method based on Lévy Flight and SA Grey Wolf Optimizer (LSAG). By integrating the global search capability of Lévy Flight and the optimization potential of SA, LSAG effectively reduces the risk of being trapped in local optima. Experimental results demonstrate that LSAG achieves notable improvements in cost optimization and convergence speed. Zhang et al. [29] developed an Enhanced Whale Optimization Algorithm (EWOA) by integrating Lévy Flight into the standard WOA. The algorithm expands the search space using the Lévy mechanism and accelerates local convergence through an adaptive crossover strategy, thereby improving optimization efficiency. Results show that EWOA outperforms other algorithms in terms of resource utilization, energy consumption, and execution cost. Zhou et al. [30] proposed a multi-objective Hybrid Artificial Bee Colony (HABC) algorithm, which introduces Lévy Flight inspired by Cuckoo Search in the onlooker bee phase to enhance search ability and address the limitations of the basic ABC algorithm in exploitation and convergence speed. Compared with four advanced Multi-Objective Evolutionary Algorithms (MOEAs), the proposed method demonstrates superior performance in multi-scale SCOS problems and achieves more competitive results. Gao et al. [31] proposed the LMPSO method, which incorporates Lévy Flight to update particles and enhance diversity. For service caching solutions, a three-stage heuristic strategy is applied for task offloading. Compared to seven other heuristic and metaheuristic algorithms, LMPSO improves user satisfaction, resource efficiency, and processing performance. Cui et al. [32] introduced DECWOA, which applies sinusoidal chaotic theory to expand the search space via a sine-based chaotic initialization process. Additionally, it incorporates adaptive inertia weights to dynamically adjust the exploration–exploitation balance, and differential variance to refine the solution space. The algorithm significantly reduces task and workflow execution time by 64% and lowers data center cost by 11%.

Advances in enhanced marine predator algorithms (EMPA) [26–32] often leverage Lévy flights and hybrid metaheuristics to strengthen search capability, yielding improvements in task scheduling and load balancing. Nevertheless, they can still face challenges such as burdensome parameter configuration, slower convergence, and elevated computational cost. Building on these insights, we fuse ASA with EMPA and introduce an adaptive factor alongside Lévy flights to balance global exploration with local intensification under explicit QoS guarantees, thereby accelerating convergence and lowering computational overhead.

## Model and formulation

### Problem description

For the hybrid cloud architecture, the core issue studied in this paper is to construct an effective scheduling strategy for the hybrid cloud resource scheduling system (hereinafter referred to as the system), so as to optimize the cost in the resource scheduling process under the premise that the QoS parameter group, namely response time (RT), end-to-end delay (D), jitter (J), and packet loss rate (PL), all meet the threshold range requirements in accordance with the industry classification standards [33]. At the same time, task resources should be allocated reasonably to achieve a balanced load of resources. Due to limitations in processing capacity, the system must fully consider the resource carrying capability to improve utilization and mitigate load imbalance. In real-world applications, the complexity of the scheduling algorithm is influenced not only by resource constraints but also by task allocation strategies in hybrid cloud environments. To address this, the system adopts an intelligent scheduling algorithm that dynamically assigns resources based on task demands and resource capabilities.

**Fig 1:**
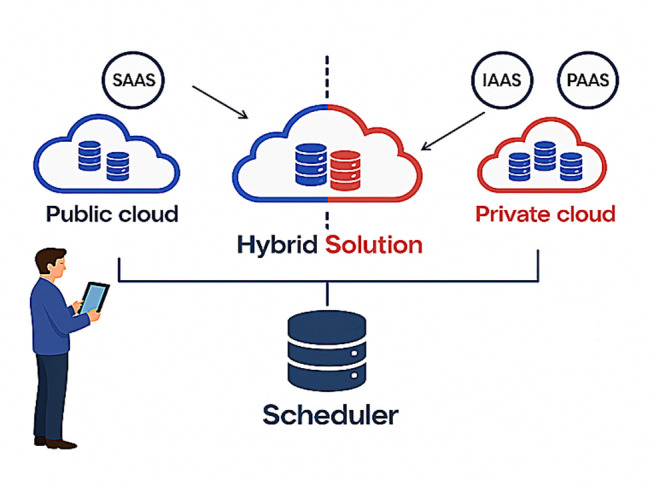
Hybrid cloud environment. The system combines private and public cloud resources and schedules tasks by evaluating computing demands, resource availability, and network communication costs. This architecture is applied under low, medium, and high load scenarios.

Taking the hybrid cloud architecture illustrated in Fig 1 as an example, the system consists of private cloud resources and multiple public cloud resources, capable of handling a large volume of user requests. To adapt to varying workloads and optimize resource utilization, the system performs intelligent scheduling upon user task submission by evaluating task computing requirements, resource availability, and network communication overhead. This ensures efficient task distribution between private and public clouds, reducing inter-cloud communication costs, balancing computational loads, and enhancing the system’s overall service capability and performance.

### Scheduling model

#### Scheduling objectives.

In a practical hybrid cloud environment, the primary goal of task scheduling is to optimize resource utilization while minimizing the overall system cost under the constraint of ensuring QoS-based user experience.

User experience should be prioritized before optimizing costs to ensure that quality of service (QoS) requirements are effectively met. A composite objective function is constructed by incorporating key parameters such as end-to-end delay, response time, jitter, throughput, and packet loss rate into the optimization model, thereby improving the overall user experience. Response time (RT) consists of the waiting time and service time experienced by tasks within the system, specifically including queueing delay and processing delay. End-to-end delay (D) refers to the network-level transmission and propagation overhead across cloud domains Throughput Th reflects the number of tasks successfully processed by the system per unit time, serving as a positive contributor to user experience. Jitter J captures the variability in end-to-end delay—greater fluctuations lead to poorer user experience. In addition, the packet loss rate PL indicates the proportion of tasks that fail during transmission, and its reduction can significantly enhance service stability.

The comprehensive user experience value is quantified through a weighted function that evaluates the impact of each parameter, as shown in Equation (1).


$$UE=\lambda \cdot \left(\frac{1}{RT}+Th\right)-\mu \cdot (J+PL+D),$$ (1)

here, response time (RT) denotes the total time from task submission to completion (queueing plus service); end-to-end delay (D) captures the network-level transmission and propagation delay; throughput (Th) reflects the number of tasks processed per unit time; jitter (J) measures the fluctuation of response time; and packet loss rate (PL) indicates the proportion of failed transmissions.

Total cost includes computing cost, storage resource cost, and communication bandwidth cost. Under the premise of meeting the user experience requirements, a reasonable task allocation strategy should be adopted to ensure efficient resource utilization and minimize the overall cost. Let the total system cost be denoted as *C*_total_. The objective function for minimizing the total cost is defined in Equation (2):


$$\min C_{\text{total}}=C_{\text{compute}}+C_{\text{storage}}+C_{\text{comm}},$$ (2)

here, *C*_compute_ denotes the cost of computing resources, *C*_storage_ represents the cost of storage resources, and *C*_comm_ refers to the cost of communication resources.

#### System state.

The system state primarily reflects the current usage of resources and the execution status of tasks. The hybrid cloud resources include both private and public cloud resources, with their states described by parameters such as CPU utilization, memory usage, and bandwidth requirements. Task states encompass computational needs, storage requirements, bandwidth needs, and QoS metrics. Here, let the system state be denoted by S, where j represents the identifier of private cloud nodes, k represents the identifier of public cloud nodes, and S consists of the following components:

Private Cloud Resource State: Each node *V*_private,*j*_ (where $j=1,2,\ldots ,M_{\text{private}}$) is located in the private cloud. Its resource usage includes CPU utilization *CPU*_private,*j*_, memory utilization *Mem*_private,*j*_, and bandwidth usage *BW*_private,*j*_ as defined in Equation (3):


$$S_{j}=\left\{CPU_{\text{private},j},Mem_{\text{private},j},BW_{\text{private},j}\right\}.$$ (3)

Public Cloud Resource State: Each node *V*_public,*k*_ ($k=1,2,\ldots ,M_{\text{public}}$) is located in the public cloud. Its resource usage includes CPU utilization (*CPU*_public,*k*_), memory utilization (*Mem*_public,*k*_), and bandwidth usage (*BW*_public,*k*_), as defined in Equation (4):


$$S_{k}=\left\{CPU_{\text{public},k},Mem_{\text{public},k},BW_{\text{public},k}\right\}.$$ (4)

Task State: Each task *T*_*i*_ (i=1,2,…,N) has an execution state that includes computing demand (*Comp*_*i*_), storage demand (*Stor*_*i*_), bandwidth requirement (*BW*_*i*_), response time (*RT*_*i*_), end-to-end delay (*D*_*i*_), jitter (*J*_*i*_), and packet loss rate (*PL*_*i*_). The task state is defined as follows in Equation (5):


$$S_{i}=\left\{{\text{Comp}}_{i},{\text{Stor}}_{i},{\text{BW}}_{i},{\text{RT}}_{i},{\text{D}}_{i},{\text{J}}_{i},{\text{PL}}_{i}\right\}.$$ (5)

#### System action.

System action represents the resource scheduling decision, i.e., how to assign tasks to nodes. Let the action set be denoted by *A*, where each action *A*_*ij*_ indicates that task *T*_*i*_ is assigned to node *V*_*j*_ or *V*_*k*_.

Task Allocation Action: Each action $A_{ij}\in A$ in *A* represents assigning task *T*_*i*_ to a node *V*_*j*_ or *V*_*k*_ in either the private or public cloud, while satisfying the corresponding resource and QoS constraints. The action is defined as follows in Equation (6):


$$a=(T_{i},V_{\text{type},j/k})\;i\in \{1,2,\ldots ,N\},\;\text{type}\in \{\text{private},\text{public}\},\;j/k\in \{1,2,\ldots ,M_{\text{type}}\},$$ (6)

here, *T*_*i*_ is one of the scheduling tasks that the system needs to assign, and the allocation must meet the resource and QoS constraints of the node. The node *V*_type,*j*/*k*_ can be either private (when type = private) or public (when type = public).

Scheduling Optimization Action: According to the feedback relationship between task-resource mappings updated by the scheduling algorithm, the task allocation is dynamically adjusted to minimize the total execution cost of the system (including computation, storage, and communication costs). The complete action set *A* can be defined as all possible task-node allocation combinations, as shown in Equation (7):


$$A=\{A_{ij}|i=1,2,\ldots ,N,\;j=1,2,\ldots ,M\}.$$ (7)

## Algorithm design (EMPA-ASA)

### Task scheduling optimization and QoS guarantee

#### Dynamic Task scheduling optimization based on MDP reinforcement learning.

In the hybrid cloud resource scheduling optimization problem, the core objective of the Markov Decision Process (MDP) is to learn an optimal policy π that maximizes the long-term cumulative reward while implicitly minimizing the operational cost. In the MDP framework, the system’s state transition is modeled by the state transition probability *P*(*s*’ | *s*, *a*), which characterizes the likelihood of the system transitioning to the next state *s*’ given the current resource and task states (as defined in Equations (3), (4), and (5)) and the applied scheduling action (as defined in Equation (7)). This transition probability during the scheduling process is formally defined in Equation (8):


$$P(s^{\prime }|s,a)=\mathrm{Pr}(S_{t+1}=s^{\prime }|S_{t}=s,A_{t}=a),$$ (8)

here, *S*_*t*_ denotes the cloud computing resource state at time *t*, including the private cloud state *S*_*j*_ and public cloud state *S*_*k*_, as well as the task execution state *S*_*i*_. *a* represents the action of assigning the current task to a specific node, that is, the task scheduling decision *A*_*ij*_. The state transition probability is jointly determined by the system’s current task load, available resource conditions, and the selected scheduling policy. The immediate reward function *R*(*s*, *a*) is used to evaluate the benefit of the current resource state and scheduling decision, and is defined as shown in Equation (9):


R(s,a)=−Cost(s,a)+QoS(s,a), (9)

here, *Cost*(*s*,*a*) represents the overall operational expenditure associated with executing action *a* under state *s*, including computing, communication, and storage consumption.

In hybrid cloud environments, the actual task completion time consists not only of service execution time but also of queueing delay caused by resource contention. To capture this temporal component, the waiting time derived from the M/M/c queueing model, denoted as *W*_*q*_ in Equation (18), is incorporated into the cost formulation as part of the effective service cost.

Accordingly, the cost function is defined as shown in Equation (10):


$$Cost(s,a)=C_{\text{comp}}(s,a)+C_{\text{comm}}(s,a)+C_{\text{stor}}(s,a)+\lambda_{d}+\lambda_{d}\sum_{r}W_{q,r}(t),$$ (10)

where $\lambda_{d}$ is a weighting coefficient that reflects the sensitivity of scheduling decisions to service response time.

The objective of the MDP is to find an optimal policy π that maximizes the expected cumulative reward under a given strategy. The expected cumulative reward under policy π is expressed in Equation (11):


$$V^{\pi }(s)=𝔼\left[\sum_{t=0}^{\infty }\gamma^{t}R(S_{t},A_{t})|S_{0}=s\right].$$ (11)

Based on this, the Bellman equation can be used to recursively compute the value function $V^{\pi }(s)$, representing the expected return from state *s*, as shown in Equation (12):


$$V^{\pi }(s)=\sum_{a}\pi (a|s)\left[R(s,a)+\gamma \sum_{s^{\prime }}P(s^{\prime }|s,a)V^{\pi }(s^{\prime })\right].$$ (12)

During the optimization process, the Q-value function *Q*(*s*, *a*) is a key element in MDP. It represents the expected cumulative reward obtained by taking scheduling action *A*_*ij*_ under the current resource state *S*, and satisfies the Bellman equation shown in Equation (13):


$$Q(s,a)=R(s,a)+\gamma \sum_{s^{\prime }}P(s^{\prime }|s,a)V^{\pi }(s^{\prime }).$$ (13)

This equation illustrates the recursive relationship of the Q-value function—namely, the value of a state-action pair not only depends on the immediate reward *R*(*s*,*a*), but also on the weighted sum of optimal value estimates under the next resource state (*S*_*j*_, *S*_*k*_) and task execution state *S*_*i*_. By iteratively updating the Q-values through repeated interactions, the system can gradually converge to the optimal policy. The optimal policy $\pi^{*}$ satisfies the following relationship, as defined in Equation (14):


$$\pi^{*}(s)=\arg\max_{a}Q(s,a).$$ (14)

Although the MDP formulation provides a rigorous theoretical framework for modeling dynamic task scheduling, directly solving the Bellman optimality equation in large-scale hybrid cloud environments is computationally prohibitive due to the exponential growth of the state–action space.

To address this issue, the MDP-based scheduling problem is reformulated as a parameterized optimization problem. Specifically, each candidate solution vector *x* encodes a feasible resource allocation strategy, which implicitly determines the corresponding state–action decisions. The cumulative reward maximization objective is equivalently transformed into the minimization of a scalar objective function Φ(x), constructed from the cost and QoS components defined in Equation (9).

Under this transformation, searching for the optimal policy $\pi^{*}$ is approximated by searching for the optimal solution vector $x^{*}$ that minimizes Φ(x). To efficiently explore the high-dimensional solution space and approximate the optimal scheduling strategy, a hybrid EMPA-ASA metaheuristic algorithm is employed in the subsequent section.

#### QoS optimization based on the M/M/c queuing model.

Grounded in quantified QoS indicators—average response time, throughput, end-to-end delay, and jitter—this work employs an *M*/*M*/*c* queuing model to optimize the end-to-end pipeline of request arrivals and parallel-server processing. Submitted tasks are then appended to a distributed message queue (RocketMQ in our implementation), while messages that timeout or exhaust their retry budget are redirected to a dead-letter queue (DLQ). This design enables cross-cloud task partitioning and dynamic load balancing via elastic adjustment of compute nodes. The response time is determined as follows: the system load factor ρ represents the load on each individual server, and is defined in Equation (15):


$$\rho =\frac{\lambda_{eff}}{c\mu }.$$ (15)

Let λ denote the arrival rate, μ the per–server service rate, and *c* the number of parallel servers. When the utilization $\rho =\lambda_{eff}/(c\mu )$ approaches 1, the system enters a congestion region in which both queue length and waiting time grow rapidly. By enabling RocketMQ-based backpressure and rate limiting, the *effective* arrival rate admitted into the queue can be reconstructed as


$$\lambda_{eff}=\min\{\lambda ,\;\theta \;c\mu \},\;0<\theta <1.$$ (16)

**Fig 2:**
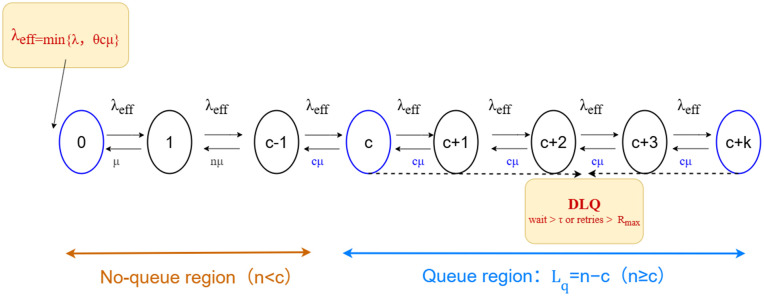
M/M/c queueing model state transition diagram. The figure illustrates task admission, queueing, service, and dead-letter queue redirection under backpressure and rate-limiting control.

Equation (16) characterizes the admission-controlled arrival rate under backpressure and rate-limiting mechanisms, where λ denotes the external task arrival rate and $\lambda_{eff}$ represents the regulated rate admitted into the processing queue. In the following queueing analysis, $\lambda_{eff}$ is treated as the effective input rate to the *M*/*M*/*c* system for evaluating load intensity and delay-related performance metrics. In addition, the no-request probability *p*_0_ represents the likelihood that the system is empty (i.e., idle). Fig 2 illustrates the state transitions and queueing semantics of the *M*/*M*/*c* model: when the number of tasks *n* < *c*, no queue forms and the *n* tasks are processed in parallel by *n* servers with a total service rate of nμ; when *n* ≥ *c*, the system enters the queueing region, the number of concurrently served tasks is capped at *c*, and the total service rate remains cμ, while the remaining *n* − *c* tasks wait in the queue (queue length *L*_*q*_ = *n* − *c*). In the figure, $\lambda_{eff}$ denotes the backpressure- and rate-limiting–shaped arrival rate; the dashed branch in the queueing region indicates tasks that are diverted to the DLQ because their waiting time exceeds the predefined business TTL τ or their retry count exceeds *R*_max_. In addition, tasks whose waiting time exceeds a predefined business TTL or whose retry count surpasses *R*_max_ are redirected to the DLQ, ensuring bounded delay and preventing persistent queue congestion.

The calculation of *P*_0_ is given by Equations (17) and (18):


$$P_{0}={\left[\sum_{n=0}^{c-1}\frac{(\lambda_{eff}/\mu {)}^{n}}{n!}+\frac{(\lambda_{eff}/\mu {)}^{c}}{c!}\cdot \frac{1}{1-\rho }\right]}^{-1},$$ (17)

$$W_{q}=\frac{P_{0}(\lambda_{eff}/\mu {)}^{c}\cdot \rho }{c!\cdot c\cdot (1-\rho {)}^{2}}.$$ (18)

The parameter *P*_0_ estimates the probability that the system remains idle under a given load condition, providing quantitative support for load management and resource provisioning. In a hybrid cloud environment, task scheduling decisions directly influence how workloads are distributed among controllers and computing nodes. As a result, both the effective arrival rate $\lambda_{eff}$ and service rate μ vary according to the selected allocation strategy. Consequently, the queueing delay *W*_*q*_ captures the response characteristics of the system under different scheduling configurations.

In a multi-controller hybrid cloud architecture, heterogeneous processing capabilities and dynamic task loads require separate evaluation of response performance for each controller. To characterize the aggregated response performance at the system level, we define the average response time metric at time slot *t*. The metric *RT*_avg_(*t*) quantifies the average response time of user requests within slot *t*, considering the resource allocation and workload distribution across both private and public cloud controllers. It is formally defined in Equation (19):


$$RT_{\text{avg}}(t)=\sum_{j\in 𝒫}\frac{Q_{j}(t)}{\mu_{j}-\lambda_{j}(t)}+\sum_{k\in 𝒢}\frac{Q_{k}(t)}{\mu_{k}-\lambda_{k}(t)},$$ (19)

here, *Q*_*j*_(*t*) and *Q*_*k*_(*t*) denote the average queue lengths of private and public cloud controllers at time slot *t*, while $\lambda_{j}(t)$ and $\lambda_{k}(t)$ represent the average task arrival rates to the private and public cloud controllers (requests per second) during slot *t*. The response time of private and public clouds is determined by their respective queue lengths *Q*_*j*_(*t*), *Q*_*k*_(*t*), and the difference between arrival and service rates. During resource scheduling, queue lengths and arrival rates of each controller are adjus*t*ed *t*o minimize the value of *RT*_avg_(*t*), thereby optimizing the system’s average response time.

Throughput indicates the number of tasks processed by the system per unit time, and is related to task arrival rate and service capacity. Based on M/M/c model parameters, the throughput is defined as shown in Equation (20):


$$T=\min(\lambda_{eff},c\cdot \mu ).$$ (20)

When the system load is low (ρ<1), throughput equals the effective arrival rate $\lambda_{eff}$; when the system is heavily loaded (ρ≥1), throughput is determined by the system’s total service capacity, i.e., c·μ.

In the M/M/c model, the response time (RT) is the sum of queueing waiting time and service time, as shown in Equation (21).


$$RT=W_{q}+\frac{1}{\mu },$$ (21)

here, *W*_*q*_ denotes the average waiting time in the queue derived from the *M*/*M*/*c* model, while 1/μ represents the average service time of a single server. Therefore, the response time RT captures both queueing delay and processing delay under the admission-controlled workload.

In addition, jitter is defined as the standard deviation of response time over a measurement window, reflecting the fluctuation of task response time. It is computed as in Equation (22):


$$J=\sqrt{\frac{1}{N}\sum_{t=1}^{N}(RT(t)-\overset{―}{RT}{)}^{2}}.$$ (22)

Through this modeling framework, the *M*/*M*/*c* analysis provides quantifiable performance indicators, including average response time, throughput, and jitter, which collectively characterize service quality under different scheduling configurations.

### EMPA-ASA resource allocation strategy

#### Solution generation in EMPA.

In this study, an enhanced Marine Predator Algorithm (EMPA) is adopted to generate new candidate solutions via Lévy flight perturbations. The Lévy flight mechanism introduces long-tailed step-size distributions, enabling dynamic search behavior in the solution space. Large perturbations facilitate global exploration across heterogeneous computing resources, thereby improving population diversity and reducing the risk of premature convergence. Conversely, smaller perturbations promote local refinement within promising regions of the search space, enhancing resource allocation precision and convergence stability. Through this adaptive perturbation behavior, EMPA effectively balances exploration and exploitation during task scheduling.

Meanwhile, the predatory behavior displacement weight $\lambda_{k}$ regulates the magnitude of the Lévy perturbation at iteration *k*. Since the temperature parameter *T*_*k*_ reflects the current search state (i.e., exploration-dominant or exploitation-dominant phase), $\lambda_{k}$ is modulated according to *T*_*k*_ to adaptively adjust the perturbation scale. At higher temperatures, larger perturbations are encouraged to enhance global exploration, whereas at lower temperatures, the perturbation magnitude is reduced to facilitate local refinement. The new solution is generated as per Equation (23):


$${\text{new}}_{\text{assignments}}^{\left(k\right)}={\text{current}}_{\text{assignments}}^{\left(k\right)}+\lambda_{k}\cdot \text{Levy}(\mu ),$$ (23)

here, $\lambda_{k}$ is the displacement weight that balances local and global search, and ${\text{current}}_{\text{assignments}}^{\left(k\right)}$ denotes the encoded solution vector representing the task–resource mapping at iteration *k* (denoted as $\Theta^{k}$ in the Objective Function subsection for notational consistency). The stochastic perturbation term Levy(μ) follows a Lévy distribution, as defined in Equation (24):


$$\text{Levy}(\mu )=\frac{u}{|v{|}^{\frac{1}{\mu }}},$$ (24)

where *u* and *v* are random numbers sampled from normal distributions, and μ is the scaling parameter of the Lévy distribution (1<μ≤3). The term $|v{|}^{\frac{1}{\mu }}$ acts as a scaling factor that stabilizes the heavy-tailed property of the Lévy distribution. By computing Levy(μ), the algorithm ensures randomness and search breadth.

#### Adaptive update mechanism.

In the SA algorithm used in this study, the temperature parameter (T) represents a key variable controlling the search range in the solution space. Its purpose is to iteratively guide the system toward the optimal resource scheduling scheme by exploring various configurations. At the early stage, a relatively high temperature is set to allow broader exploration across the solution space, thereby improving global search capability. As the search proceeds, T is gradually reduced through a local annealing process. When T converges, the algorithm becomes less likely to accept inferior solutions, shifting the strategy from global exploration to fine-tuned local optimization—ultimately identifying an optimal scheduling scheme that satisfies QoS constraints.

To support this process, we introduce an adaptive factor *g*_*k*_ into the traditional simulated annealing framework. This factor dynamically adjusts the cooling rate based on real-time changes in solution quality. The calculation of *g*_*k*_ is defined in Equations (25)–(29):


$$g_{k}=h_{1}\cdot \Delta f+h_{2}\cdot \left(R_{\text{target}}-R_{k}\right)+h_{3}\cdot \left(\Delta N_{\text{target}}-\Delta N_{k}\right),$$ (25)

here, Δf denotes the relative change of the composite objective value between the newly generated solution and the current solution, reflecting the fluctuation in solution quality:


$$\Delta f=\frac{\left|\Phi_{new}-\Phi_{current}\right|}{\Phi_{current}}.$$ (26)

The term (*R*_target_ − *R*_*k*_) reflects the difference in acceptance rate to determine whether improvements are being made, and characterizes the quality fluctuation of the solution:


$$R_{k}=\frac{\text{accepted solutions}}{\text{total solutions}}.$$ (27)

The neighborhood variation $\left(\Delta N_{\text{target}}-\Delta N_{k}\right)$ determines whether suboptimal solutions should be accepted, thereby preventing premature convergence to local optima. The value $\Delta N_{k}$ is calculated as:


$$\Delta N_{k}=\frac{1}{d}{\left\|\Theta^{k}-\Theta^{k,new}\right\|}_{1},$$ (28)

where ${𝐬}^{\left(k\right)}\in {\mathbb{R}}^{d}$ represents the assignment state vector at iteration *k*, *d* denotes the dimensionality of the solution space, and $\|\cdot {\|}_{1}$ is the L1 norm measuring the difference between two neighboring states. Each component of the adaptive factor is weighted by coefficients *h*_1_, *h*_2_, and *h*_3_, which determine their influence on the temperature evolution during the search process. Based on this adaptive control mechanism, the temperature parameter is updated as follows:


$$T_{k+1}=T_{k}\times (1+\beta g_{k}).$$ (29)

To ensure numerical stability, the adaptive factor *g*_*k*_ is restricted within a predefined bounded interval before the temperature update. This constraint prevents excessive temperature fluctuations and guarantees that *T*_*k*_ remains positive during the iterative process.

Within the adaptive simulated annealing (ASA) framework, the temperature *T*_*k*_ determines the acceptance behavior of newly generated candidate solutions. According to the classical Metropolis criterion, an inferior solution is accepted with probability


$$P_{\text{accept}}=\exp\left(-\frac{\Delta \Phi_{k}}{T_{k}}\right),\;\text{if }\Delta \Phi_{k}>0,$$ (30)

where $\Delta \Phi_{k}$ denotes the objective variation between the candidate solution and the current solution at iteration *k*.

By incorporating the acceptance mechanism defined in Eq. (31) into the Lévy-based perturbation process described in Eq. (23), we obtain the integrated update equation of EMPA-ASA, which governs the transition of the decision vector from one iteration to the next. The objective variation $\Delta \Phi_{k}$ required in this process is first defined as:


$$\Delta \Phi_{k}=\Phi (\Theta^{k,new})-\Phi (\Theta^{k}),$$ (31)

where Φ(Θ) represents the composite cost–QoS objective value associated with decision vector Θ.

The stochastic update rule of the decision vector is then given by


$$\begin{array}{c} \Theta^{k+1}=\left\{ \begin{array}{cc} \Theta^{k,new}, & \Delta \Phi_{k}\leq 0,\\ \Theta^{k,new}, & \text{with probability }\exp\left(-\frac{\Delta \Phi_{k}}{T_{k}}\right),\\ \Theta^{k}, & \text{otherwise}. \end{array}\right. \end{array}$$ (32)

In Eq. (32), $\Theta^{k,new}$ is generated according to the Lévy perturbation rule in Eq. (23), while the temperature *T*_*k*_ evolves adaptively through Eq. (29). The state transition of Θ is therefore determined by the perturbation mechanism together with the temperature-dependent acceptance rule during the iterative process. This update rule describes how the algorithm iteratively explores the solution space, combining Lévy-based exploration with adaptive local search via the Metropolis acceptance criterion.

**Fig 3:**
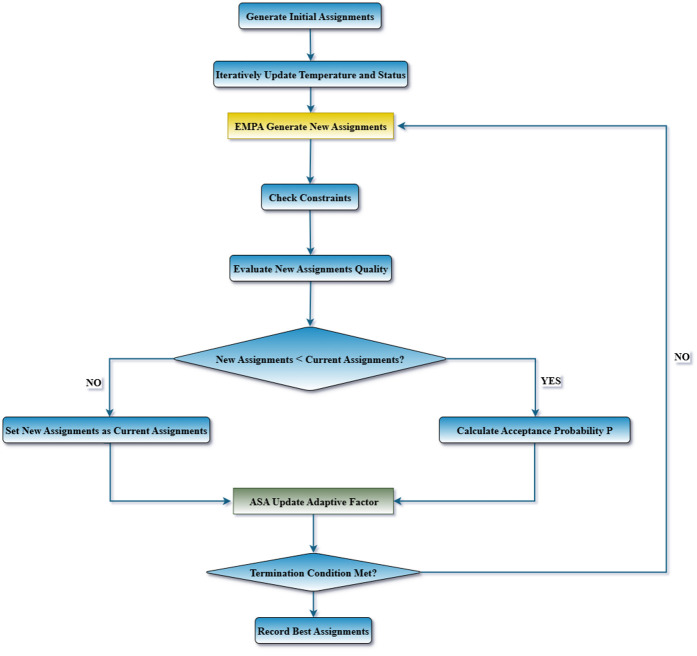
EMPA generate new assignments and ASA adaptive factor update. This figure illustrates the overall process of generating a new solution in EMPA. The Levy flight step size is adaptively adjusted according to the ASA adaptive factor to balance global exploration and local exploitation.

The overall process of generating new solutions in EMPA, integrated with the adaptive update factor *g*_*k*_, is illustrated in Fig 3.

### Objective function

To jointly consider economic efficiency and service quality, the overall objective is formulated as a penalty-based single-objective model. The total operational cost component is first defined as follows:


$$C_{compute}=\sum_{i\in \{private,\;public\}}(\alpha_{i}\cdot {CPU}_{i}(t)+\beta_{i}\cdot {Mem}_{i}(t)),$$ (33)

where $\alpha_{i}$ and $\beta_{i}$ denote the computational cost coefficients of the private and public clouds, respectively, and CPU_*i*_(*t*) and Mem_*i*_(*t*) represent the CPU and memory utilization at time *t*.

The optimization decision vector of this work is defined as $\Theta =\{x_{i,s}(t),a_{i}(t),c_{r}(t)\}$, where $x_{i,s}(t)\in \{0,1\}$ indicates whether task *i* is assigned to server *s*, satisfying $\sum_{s}x_{i,s}(t)=1$. Here $a_{i}(t)\in \{0,1\}$ is the admission variable, and $c_{r}(t)\in {\mathbb{Z}}_{\geq 1}$ represents the number of parallel servers. To ensure consistency with realistic system load levels, the parameters are constrained as $c_{r}^{\min}\leq c_{r}(t)\leq c_{r}^{\max}$, $\lambda_{i}^{\min}\leq \lambda_{i}(t)\leq \lambda_{i}^{\max}$, and $\mu_{r}>0$. The resource quantities satisfy $0\leq {CPU}_{i}(t)\leq {CPU}_{i}^{\max}$, $0\leq {Mem}_{i}(t)\leq {Mem}_{i}^{\max}$, $0\leq {Sto}_{i}(t)\leq {Sto}_{i}^{\max}$, and $0\leq {BW}_{i}(t)\leq {BW}_{i}^{\max}$. All cost coefficients $\alpha_{i},\beta_{i},K^{comp},K^{eq}>0$. The effective arrival rate assigned to resource *r* after task admission is given in Equation (34):


$$\Lambda_{r}(t)=\sum_{i\in 𝒯}a_{i}(t)\;x_{i,r}(t)\;\lambda_{i}(t).$$ (34)

For the corresponding *M*/*M*/*c*_*r*_(*t*) queue, the traffic intensity coefficient is $\rho_{r}(t)=\Lambda_{r}(t)/[c_{r}(t)\mu_{r}]$. Let *P*_*W*,*r*_(*t*) denote the Erlang–C probability of wai*t*ing in the queue. The average waiting time is expressed in Equation (35):


$$W_{q,r}(t)=\frac{P_{W,r}(t)}{c_{r}(t)\mu_{r}-\Lambda_{r}(t)},\;R_{r}(t)=W_{q,r}(t)+\frac{1}{\mu_{r}}+\ell_{r},$$ (35)

where $\ell_{r}$ denotes the link propagation delay. The response time at resource *r* is therefore $R_{r}(t)=W_{q,r}(t)+\ell_{r}+s_{r}(t)$, where *s*_*r*_(*t*) represents the service time. For a task *i* assigned to resource *r* (with binary indicator *x*_*i*,*r*_(*t*) satisfying $\sum_{r}x_{i,r}(t)=1$), its overall response time is $R_{i}(t)=\sum_{r}x_{i,r}(t)\;R_{r}(t)$, and the jitter upper bound *J*_*i*_(*t*) is compu*t*ed accordingly.

To regulate service quality, the following QoS conditions define the acceptable performance bounds: $R_{i}(t)\leq R_{i}^{\max}$ (response time constraint), $J_{i}(t)\leq J_{i}^{\max}$ (jitter constraint).

The system stability and admission constraints are given in Equation (36):


$$\Lambda_{r}(t)\leq \Gamma_{r}(t)\leq c_{r}(t)\mu_{r},\;\sum_{i}(1-a_{i}(t))\leq {DLQ}^{\max}(t),$$ (36)

where $\Gamma_{r}(t)$ denotes the permissible service rate dynamically provisioned for server pool *r*, and ${DLQ}^{\max}(t)$ is the upper bound of tasks temporarily redirected to the dead-letter queue.

The total operational cost is defined as Equation (37):


$$C_{total}=C_{compute}+C_{storage}+C_{communication},$$ (37)

where *C*_compute_, *C*_storage_, and *C*_communication_ correspond to the costs of computation, storage, and network transmission, respectively. Accordingly, the objective of the proposed EMPA–ASA algorithm is to minimize the composite objective Φ(t), which integrates total cost and QoS violation penalties, thereby achieving a balanced trade-off between performance and economic efficiency. In the subsequent optimization process, the system-level decision vector $\Theta =\{x_{i,s}(t),a_{i}(t),c_{r}(t)\}$ is iteratively updated. For notational simplicity in Algorithm 1, the solution at iteration *k* is denoted as $\Theta^{k}$, and the candidate solution generated at iteration *k* is denoted as $\Theta^{k,new}$. Therefore, the optimization variables manipulated in the algorithm directly correspond to the decision variables defined in the objective model above.

To jointly consider economic efficiency and service quality, we construct the composite objective function as (38)


$$\begin{array}{cc} \Phi (t)=C_{total}(t)+\sum_{i}[ & \eta_{1}\max(0,R_{i}(t)-R_{i}^{\max})\\ & +\eta_{2}\max(0,J_{i}(t)-J_{i}^{\max})], \end{array}$$ (38)

where $\eta_{1},\eta_{2}>0$ are penalty coefficients that quantify the importance of QoS violations.

**Fig 4:**
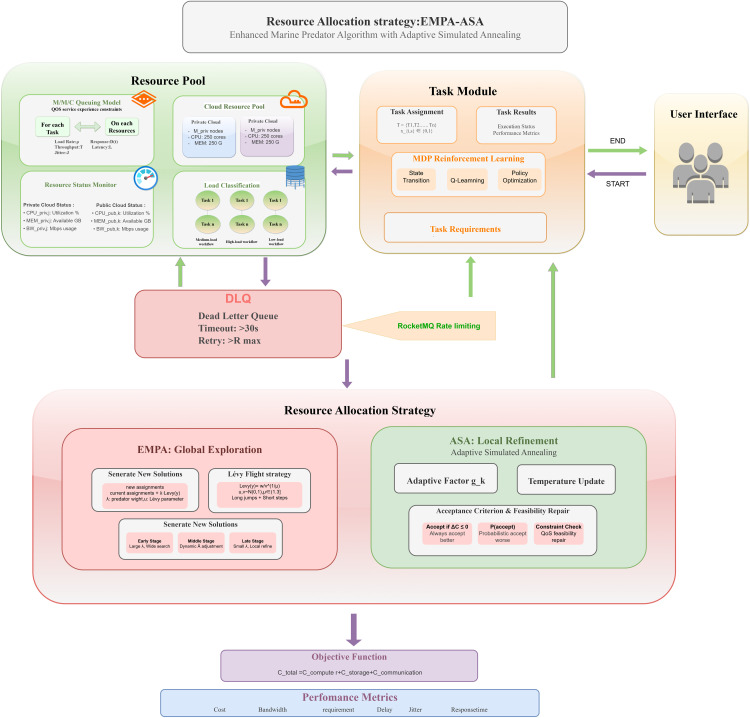
EMPA-ASA resource scheduling optimization process. The figure illustrates the optimized resource scheduling workflow of EMPA-ASA, which is divided into two main components: the task module and the resource module.

Fig 4 presents the EMPA-ASA resource–scheduling optimization workflow. On the task side, an MDP-based reinforcement learning method produces the initial task assignment under QoS constraints. On the resource side, tasks are first categorized into load levels (low, medium, high) [34] and submitted to the cloud resource pool for allocation and processing. An *M*/*M*/*c* queueing model is then used to determine whether to trigger admission and backpressure. The resource-limitation module evaluates each task’s QoS requirements and continuously monitors the status of all resources. Subsequent tuning proceeds according to real-time service capacity: when load drops, a resource-contraction notice is issued; at this point, RocketMQ enforces admission and backpressure based on current service capability, redirecting tasks that exceed the timeout or retry limit to the DLQ and releasing surplus resources to cut computational overhead. When a resource bottleneck or overload is detected, an expansion notification is sent to the user side and elastic scaling is performed as needed. If scaling is infeasible, the system still applies admission and backpressure, redirects timeout/over-retry tasks to the DLQ, and removes the expansion restriction once the load recedes. The optimized queue and resource states are refined by EMPA-ASA: EMPA conducts global exploration, while ASA iterates with an adaptive factor and temperature *T*. The scheduling decisions are fed back to the task side as the next MDP state for policy update, forming a dynamic cycle of”monitoring – admission/back pressure – scheduling optimization – execution – feedback”. The pseudocode of EMPA-ASA is given in Algorithm 1.

**Algorithm 1: The EMPA-ASA Algorithm**

**Input:** Task set 𝒯, Resource set ℛ, QoS parameters, MaxIter, *T*_0_

**Output:** Optimal decision vector $\Theta^{*}$ minimizing composite objective

1.   Initialize iteration counter *k* = 0.

2.   Initialize decision vector $\Theta^{0}$ and set $\Phi_{current}=\Phi (\Theta^{0})$ according to Eq. (38).

3.   Set temperature *T*_*k*_ = *T*_0_, initialize displacement weight $\lambda_{k}=\lambda_{0}T_{k}$.

4.   Initialize BestSolution = $\Theta^{0}$, $\Phi_{best}=\Phi_{current}$, StagnationCount = 0.

5.   **while** $T_{k}>T_{\min}$ and *k* < *MaxIter* **do**

6.   Generate candidate solution using Lévy perturbation Eq. (23): $\Theta^{k,new}=\Theta^{k}+\lambda_{k}\cdot \text{Levy}(\mu )$.

7.    Apply feasibility projection to satisfy constraints in Eqs. (34)–(36).

8.   Evaluate queueing metrics using Eqs. (34)–(35) and compute objective value $\Phi_{new}=\Phi (\Theta^{k,new})$.

9.   Compute objective variation using Eq. (31): $\Delta \Phi_{k}=\Phi_{new}-\Phi_{current}$.

10.   Compute acceptance probability according to Eq. (30): *P* = 1 if $\Delta \Phi_{k}\leq 0$, otherwise $P=\exp\left(-\frac{\Delta \Phi_{k}}{T_{k}}\right)$.

11.   **if** accepted with probability *P* **then**

12.     $\Theta^{k+1}=\Theta^{k,new}$.

13.     $\Phi_{current}=\Phi_{new}$.

14.   **else**

15.     $\Theta^{k+1}=\Theta^{k}$.

16.   **end if**

17.   Update best solution:

18.     **if** $\Phi_{current}<\Phi_{best}$ **then**

19.       BestSolution = $\Theta^{k+1}$.

20.       $\Phi_{best}=\Phi_{current}$.

21.       StagnationCount = 0.

22.     **else**

23.       StagnationCount = StagnationCount + 1.

24.     **end if**

25.   Compute adaptive factor *g*_*k*_ using Eqs. (25)–(28).

26.   Update temperature according to Eq. (29): $T_{k+1}=T_{k}(1+\beta g_{k})$.

27.   Update displacement weight: $\lambda_{k+1}=\lambda_{0}T_{k+1}$.

28.   *k* = *k* + 1.

29. **end while**

30. **return** BestSolution.

## Experiment and simulation

The experimental scenarios are based on a unit where the client is located. The performance evaluation metrics of the experiment are based on user experience, mainly analyzed from two aspects: First, the user’s computing capability, including end-to-end delay, jitter, response time, and whether the QoS requirements are met; second, the ability to optimize resource allocation costs, including a comprehensive comparison of computing, storage, and communication costs. The experiment finally evaluates the algorithm’s practical application value by comparing the results of the resource optimization agreed upon by the user. The simulation experiments are conducted in a Python language environment, where the proposed EMPA-ASA algorithm is compared with the following algorithms: GA, PSO, SA, and EMPA algorithm. Each algorithm is run for 1000 iterations with a population size of 100. Each algorithm is executed 50 times, and the results are averaged for comparison.

Experiments are carried out on a hybrid-cloud simulation platform, with the private-cloud controller provisioned by virtualizing a local host, and the public-cloud controller provisioned through virtualizing a remote cloud server. The system configuration includes 2 CPU cores (1 core = 8 CPUs) and 32 GB of memory for the private cloud, and 4 CPU cores with 64 GB of memory for the public cloud. The inter-cloud network provides 1 Gbps bandwidth with a round-trip time (RTT) of 10–20 ms. Both the algorithm and evaluation toolkit are implemented in Python 3.12. The baselines include GA, PSO, SA, and EMPA, which are selected as representative optimization algorithms for hybrid-cloud resource scheduling. GA and PSO are widely adopted in cloud and hybrid-cloud optimization problems due to their established ability to balance exploration and exploitation. They provide a strong foundation for comparison by demonstrating the general capabilities of evolutionary and swarm-based optimization techniques. SA, while a classical approach, serves as a baseline for evaluating local search efficiency, while EMPA is chosen to validate the unique contributions of the proposed ASA-enhanced strategy. To ensure fairness, all methods are run with the same settings of 1,000 iterations and a population size of 100, executed 50 times with different random seeds. The results are averaged and presented with 95% confidence intervals.

In accordance with the ITU–T G.1010 QoS guideline [35], and combined with the practical deployment context of our partner site, we set the relevant parameters and symbols as summarized in Table 1. To strengthen reproducibility, Table 1 lists only the key hyperparameters and uses a uniform experimental configuration unless otherwise noted: for Q-learning, the discount factor is set to γ=0.8 to balance short- and long-term rewards, and the learning rate adopts a time-decay schedule with initial value α=0.1 to improve convergence stability. For EMPA-ASA, the adaptive factor is initialized to ω=0.5 and is automatically adjusted online according to the acceptance rate so as to balance global exploration and local intensification. Unless specified, the initial search budget is fixed at 1,000 iterations with a population size of 100.

**Table 1:** Parameters, symbols, and values.

Parameter	Symbol	Value
Number of Private Cloud Controllers	*M*_*priv*_	90
Number of Public Cloud Controllers	*M*_*pub*_	100
Private Cloud CPU Capacity	*CPU*_*priv*_	250 cores (1 core = 8 CPUs)
Public Cloud CPU Capacity	*CPU*_*pub*_	500 cores (1 core = 8 CPUs)
Private Cloud Memory Capacity	*MEM*_*priv*_	250 GB
Public Cloud Memory Capacity	*MEM*_*pub*_	500 GB
Private Cloud Service Rate	*S*_*priv*_	10 requests/sec
Public Cloud Service Rate	*S*_*pub*_	5 requests/sec
Maximum Response Time	*T*_*response*_	10 seconds
Maximum end-to-end delay	*L*_*max*_	300 ms
Maximum Bandwidth	*B*_*max*_	1000 GB
Maximum Jitter	*J*_*max*_	150 ms
Maximum Packet Loss	*P*_*loss*_	0.3%
Minimum Throughput	*T*_*throughput*,*min*_	3 GB/s
Maximum Throughput	*T*_*throughput*,*max*_	10 GB/s
Adjustment Factor	ω	0.5
Discount Factor	γ	0.9
Learning Rate	α	0.1
Maximum Completion Time	*T*_*completion*_	60 seconds
Maximum Iterations	*T*_*max*_	100
Timeout	*T*_*timeout*_	30 seconds
Minimum User Experience Threshold	*T*_*min*_	0.00001

In addition, we simulate network performance under low, medium, and high loads, with parameter ranges summarized in Table 2. The load-specific parameters in Table 2 are preset to emulate demand under different network-protocol scenarios. *Low load* primarily corresponds to lightweight HTTP applications (e.g., web browsing, file transfer, email), where bandwidth consumption is modest and both end-to-end delay and jitter remain within a small range. *Medium load* is dominated by HTTPS downloads and batch transfers, routine API/database interactions, and generally balanced traffic; end-to-end delay and jitter stay within acceptable bounds, and resource utilization reaches a moderate level. *High load* arises in large-scale data movement and real-time interactive services—such as cloud computing, telemedicine, and video conferencing—where the network approaches saturation, end-to-end delay and jitter increase markedly, and the system faces stricter optimization requirements.

**Table 2:** Performance parameters under different loads.

Parameters	Low load	Medium load	High load
Bandwidth requirement (Mbps)	0–2000	2000–5000	5000–10000
Delay (ms)	0 ~ 50	50 ~ 100	100 ~ 300
Jitter (ms)	0 ~ 5	5 ~ 20	20 ~ 150
Response time (s)	0 ~ 0.3	0.3 ~ 0.5	0.5 ~ 10

**Fig 5:**
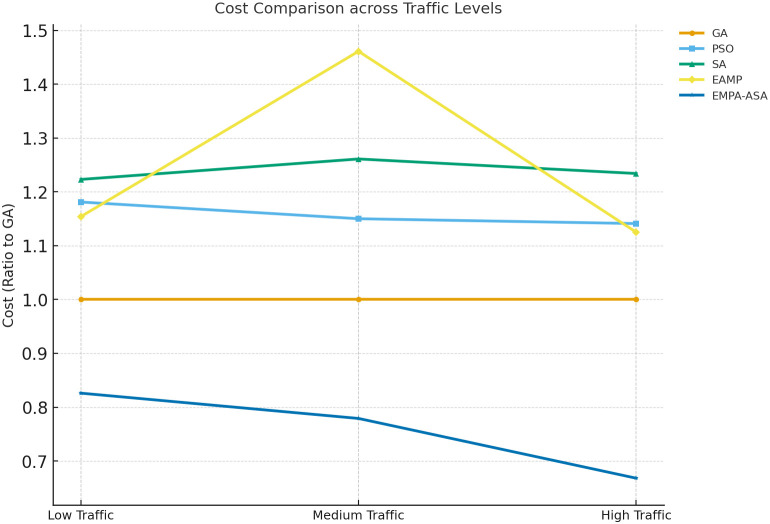
Cost comparison of different algorithms. Total cost comparison of EMPA-ASA against GA, PSO, SA, and EMPA under low, medium, and high load conditions. Costs include computing, storage, and communication.

**Fig 6:**
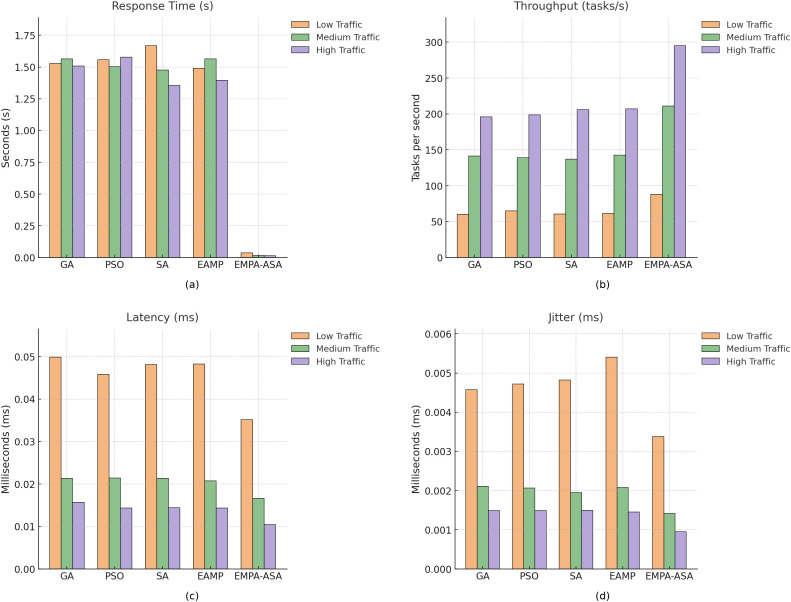
Comparison of performance metrics across different algorithms under varying traffic loads. **(a)** Response time; **(b)** Throughput; **(c)** End-to-end delay; **(d)** Jitter.

Cost comparisons under different loads are reported in Fig 5. Using GA as the baseline (normalized cost = 1), EMPA-ASA achieves the lowest total cost for low, medium, and high loads alike; notably, under the medium-load condition its total cost is reduced by approximately 48% and 70% relative to GA and PSO, respectively. All reported results are obtained from 50 independent runs with different random seeds, and the averaged values are used for comparison. Statistical tests indicate that the overall differences among the three load regimes are significant (*p* < 0.05), and all four QoS indicators show clear advantages in Fig 6. The statistical analysis is conducted using one-way ANOVA and the Friedman test with a significance level of α=0.05; when overall significance is detected, post-hoc comparisons are further performed to identify pairwise differences among algorithms. Aggregating across low/medium/high scenarios, EMPA-ASA significantly outperforms the competitors in response time, throughput, and jitter (*p* < 0.05), with the largest gains observed at high load. Detailed numerical results for the high-load case are provided in Table 3.

**Table 3:** The performance comparison between the proposed algorithm and other algorithms.

Reference	Method	Cost (Ratio)	Response Time (s)	Throughput (tasks/s)	End-to-end Delay (ms)	Jitter (ms)	Statistical Significance
Chen Q H et al. [36]	GA	1.000	1.50	195.33	0.0157	0.001493	**
Dornala R R et al. [37]	PSO	1.141	1.57	198.59	0.0149	0.001490	**
Lv K et al. [24]	SA	1.234	1.35	205.82	0.0144	0.001499	*
Gong R et al. [26]	EMPA	1.125	1.39	210.02	0.0140	0.001451	*
**Our proposed**	**EMPA-ASA**	**0.668**	**0.01**	**294.74**	**0.0104**	**0.000952**	—

Each result is averaged over 50 independent runs. Statistical significance is evaluated at a significance level of α=0.05 using one-way ANOVA and the Friedman test. Symbols * and ** denote significant differences with respect to the proposed EMPA-ASA method based on post-hoc comparisons (*p* < 0.05).

As seen in Fig 6, EMPA-ASA exhibits consistent superiority across the four QoS metrics under all three load conditions. First, in terms of response time, EMPA-ASA attains the minimum response latency across all loads, reaching as low as 0.01 s at high load, thereby improving system responsiveness. This improvement directly reflects the effectiveness of the response-time constraint incorporated in the optimization model, which prioritizes timely task execution under varying workloads. Second, throughput is also outstanding: in all scenarios EMPA-ASA surpasses the other methods, with the high-load peak reaching 294.74 tasks/s, outperforming classical EMPA and Simulated Annealing. This behavior is consistent with the throughput-related QoS constraint, which aims to maintain sufficient processing capacity and avoid performance degradation as the load increases. Third, end-to-end delay and queueing delay decrease with increasing load because tasks are dynamically distributed across servers, which reduces single-queue buildup and processing delay; under high load, response time jitter remains highly stable, with the minimum observed value as low as 0.00095 s, indicating robust temporal smoothness even near saturation. The observed stability in jitter demonstrates that the jitter constraint effectively limits performance fluctuations caused by dynamic resource contention, even under near-saturation conditions.

For inferential statistics, each metric is evaluated over 50 independent runs per load level. We first test normality and homoscedasticity; if both assumptions hold, we apply one-way ANOVA [38] to assess overall differences and report the effect size ($\eta^{2}$). When assumptions are violated, we instead use the Friedman test. Post-hoc comparisons follow a Tukey HSD procedure (after ANOVA) or a Nemenyi test (after Friedman), with Holm–Bonferroni correction for multiple comparisons. In Table 3, pairwise results versus EMPA-ASA are marked with asterisks in the “Significance vs. EMPA-ASA” column.

**Fig 7:**
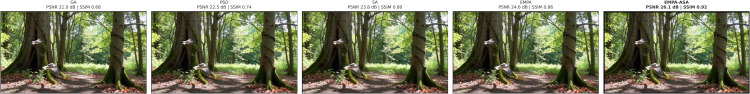
Visual quality and normalized time-complexity across scheduling algorithms in a 1080p video-conference scenario. 1920×1080, H.264, 4 Mb/s, GOP = 50.

With total cost already minimized and QoS unaffected, we further verify the QoS effectiveness of different schedulers in a business scenario using a high-definition video–conference emulation. Under the same video source and network conditions, five algorithms—GA, PSO, SA, EMPA, and EMPA-ASA—are used for scheduling control. The video parameters are: 1920×1080 resolution, H.264 codec, bitrate 4 Mb/s, and GOP = 50. For each method, received frames at identical timestamps are extracted for side-by-side comparison (see Fig 7), and the corresponding PSNR and SSIM metrics are reported. The results show that GA and PSO produce reconstructed frames with noticeable blur and blocking artifacts; SA and EMPA improve detail preservation; in contrast, EMPA-ASA achieves the best overall sharpness and structural consistency, with the highest PSNR and SSIM values among all methods.

**Fig 8:**
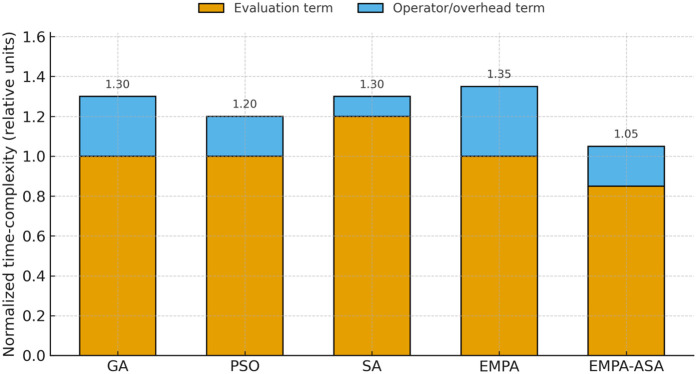
Comparison of normalized computational time complexity under a unified budget. Each bar is split into the *evaluation component* ($\approx G\;\cdot \;P\;\cdot \;C_{eval}$; for SA: $G_{s}\;\cdot \;N_{nb}\;\cdot \;C_{eval}$) and the *operator/overhead component* (≈G·P·D; for SA: $G_{s}\;\cdot \;D$). All values are normalized with respect to GA’s evaluation component as the baseline (=1).

Under identical iterations and population size, the computational time–complexity comparison is shown in Fig 8. With GA’s *evaluation component* normalized as the baseline (value = 1), EMPA-ASA attains the lowest total complexity; both its evaluation and operator/overhead components are smaller than or comparable to those of the baselines, indicating fewer effective evaluations and shorter wall-clock time under the same budget. This demonstrates that EMPA-ASA achieves higher convergence efficiency and computational economy while preserving QoS.

Based on the above experimental results, it can be seen that the EMPA-ASA algorithm outperforms existing methods in several key metrics, including task allocation cost, system delay, jitter, throughput, and bandwidth utilization. Particularly in high-load scenarios, EMPA-ASA demonstrates strong adaptability and QoS control capabilities, providing a more efficient and stable solution for hybrid cloud resource allocation.

In practical applications, the system can dynamically adjust itself according to changes in task scale: 1) Under normal conditions, the system dynamically monitors the queue length and task arrival rate through the RocketMQ component, and promptly adjusts the effective arrival rate; 2) In a congested state, that is, when the system detects that the service capacity is approaching the QoS boundary value, it applies to add new nodes to enhance the service capacity, while the RocketMQ component temporarily limits the entry rate of new tasks to prevent excessive queue backlog and uncontrollable queue queuing delay. 3) After expansion is completed, the system returns to the state of 1). During this process, the update strategy of EMPA-ASA based on the integration of Lévy flight and adaptive simulated annealing remains unchanged, and the algorithm complexity does not show a significant increase. In terms of convergence, from state 1) to state 2), due to the backpressure mechanism temporarily limiting the task inflow, the system load pressure is relieved, and the algorithm iteration can still maintain the original convergence trend; from state 2) to state 3), after expansion, the system processing capacity is improved, the task queue gradually clears, and after moderately increasing the number of iterations, the algorithm quickly recovers the convergence efficiency, and ultimately maintains the original convergence accuracy.

## Conclusion

This paper systematically explores the core challenges faced by efficient resource scheduling in a hybrid cloud environment. In view of the limitations of existing algorithms in parameter optimization, QoS index optimization, and high computing costs, an innovative hybrid cloud resource scheduling algorithm is proposed. The algorithm effectively tackles these challenges by integrating dynamic congestion control and load-aware adaptation through MDP and Q-learning, enabling real-time policy adjustments. Additionally, it incorporates elastic resource allocation via admission and backpressure mechanisms, which ensures system performance is maintained even under varying loads. Finally, the hybrid EMPA–ASA optimization approach is employed to balance global search with local intensification, optimizing both QoS and cost. The simulation experiments and comparative analysis results show that, compared with the baseline algorithm, this algorithm performs well in comprehensive indicators and can significantly improve resource utilization and reduce total costs while ensuring QoS. This empirically verifies the effectiveness and superiority of integrating this algorithm into hybrid cloud scheduling management under dynamic loads. Although this research has achieved the aforementioned expected results, its current validation is limited to a simulated environment, and the range of QoS indicators used is limited. Moreover, it does not include a comprehensive security coordination mechanism. Future work will therefore focus on three aspects: 1) deploying the algorithm in large-scale production environments involving multiple cloud providers to validate its performance under real-world dynamic loads; 2) extending the QoS evaluation framework to include additional dimensions such as SLA violation rate, resource fragmentation, and energy consumption; and 3) developing a unified security orchestration mechanism that integrates fine-grained access control, ciphertext computing, and privacy preservation into the scheduling process.

## Supporting information

S1 File Source data and implementation files for hybrid cloud resource scheduling. This file contains the source data and implementation files used in this study.
(ZIP)
